# On Scalability of FDD-Based Cell-Free Massive MIMO Framework

**DOI:** 10.3390/s23156991

**Published:** 2023-08-07

**Authors:** Beenish Hassan, Sobia Baig, Saad Aslam

**Affiliations:** 1 Department of Electrical Engineering, The Islamia University of Bahawalpur, Bahawalpur 63100, Pakistan; benish.hassan@iub.edu.pk; 2 Department of Electrical and Computer Engineering, Energy Research Center, COMSATS University Islamabad, Lahore Campus, Lahore 54000, Pakistan; drsobia@cuilahore.edu.pk; 3 Department of Computing and Information Systems, School of Engineering and Technology, Sunway University, Petaling Jaya 47500, Selangor, Malaysia

**Keywords:** cell-free, massive MIMO, scalable FDD, angular reciprocity, dynamic cooperation clustering

## Abstract

Cell-free massive multiple-input multiple-output (MIMO) systems have the potential of providing joint services, including joint initial access, efficient clustering of access points (APs), and pilot allocation to user equipment (UEs) over large coverage areas with reduced interference. In cell-free massive MIMO, a large coverage area corresponds to the provision and maintenance of the scalable quality of service requirements for an infinitely large number of UEs. The research in cell-free massive MIMO is mostly focused on time division duplex mode due to the availability of channel reciprocity which aids in avoiding feedback overhead. However, the frequency division duplex (FDD) protocol still dominates the current wireless standards, and the provision of angle reciprocity aids in reducing this overhead. The challenge of providing a scalable cell-free massive MIMO system in an FDD setting is also prevalent, since computational complexity regarding signal processing tasks, such as channel estimation, precoding/combining, and power allocation, becomes prohibitively high with an increase in the number of UEs. In this work, we consider an FDD-based scalable cell-free network with angular reciprocity and a dynamic cooperation clustering approach. We have proposed scalability for our FDD cell-free and performed a comparative analysis with reference to channel estimation, power allocation, and precoding/combining techniques. We present expressions for scalable spectral efficiency, angle-based precoding/combining schemes and provide a comparison of overhead between conventional and scalable angle-based estimation as well as combining schemes. Simulations confirm that the proposed scalable cell-free network based on an FDD scheme outperforms the conventional matched filtering scheme based on scalable precoding/combining schemes. The angle-based LP-MMSE in the FDD cell-free network provides 14.3% improvement in spectral efficiency and 11.11% improvement in energy efficiency compared to the scalable MF scheme.

## 1. Introduction

Fifth-generation systems promise to provide data rates as much as ten times higher compared to the previous generations of wireless communication, and that too at a faster pace [1,2]. The utilization of millimeter wave bands makes it possible to achieve the goals set in terms of data rate and latency. However, there are some challenges for millimeter wave communications which require high-performance estimation schemes with lower computational complexity as well as efficient interference management and optimal choice of duplexing scheme [3,4]. Possible solutions may include the utilization of massive MIMO, the use of suitable cooperation mechanisms such as cell-free framework, and deployment of optimal estimation and precoding/combining schemes [4,5].

The concept of the cell-free massive MIMO has the potential to greatly reduce interference of cellular networks [6]. Since cell-free frameworks have a large coverage area, they are inherently equipped with diversity gains, thus providing immunity against fading and shadowing effects [7]. However, the cell-free framework requires a huge amount of control signaling with the core network and increased backhaul requirements. The key feature of cell-free is to create a network, where all the APs cooperate in order to jointly serve the UEs and eliminate inter-cell interference [8,9]. At the core of the network, there is a central processing unit (CPU), and all APs in the network are connected to it through the backhaul link [10,11].

Performance of the cell-free framework relies on the acquisition of accurate channel state information (CSI) statistics [9]. These CSI estimates are required for precoding during transmission and interference mitigation in the spatial domain. Acquiring these estimates requires sending/receiving pilots, which subsequently incurs greater overhead as well as computational complexity [9,10]. Channel estimation techniques may result in high computational complexity for cell-free frameworks [11,12]. Since the utilization of channel reciprocity in time division duplexing (TDD) requires only estimating CSI in the uplink, therefore, pilot overhead and estimation cost are reduced [4]. Frequency division duplex (FDD) is another duplexing mode that can offer equal improvement in terms of reduced pilot overhead and computational complexity. Although the FDD does not possess channel reciprocity, it has angular reciprocity where angles of departure (AoDs) are the same in both uplink and downlink [5]. The utilization of a TDD protocol is suitable in a sub-6 GHz band, due to time reciprocity. However, an FDD can leverage from a millimeter wave frequency band since angular reciprocity is valid for larger frequency ranges [4,5]. In addition, FDD-based wireless systems still dominate wireless communication due to their lower cost and better coverage compared to their TDD counterparts [5].

The scalability of an FDD cell-free network is another challenge that is based on AP’s ability to process and share data in a computationally efficient manner [4]. The complexity of these tasks increases linearly with the increase in the number of UEs [13]. To overcome this issue in an FDD cell-free framework, it is imperative to acquire some kind of adaptive cooperative clustering scheme that can provide a scalable network operation, as suggested by authors in [4,13]. In addition, an appropriate algorithm for power control is also required in a cell-free network to address the scalability with the increasing number of users joining the network [4,5]. It is worth mentioning here that all APs make independent power allocation decisions, not knowing the power allocation at the neighboring APs, therefore, scalable solutions are difficult to devise. A heuristic approach to the power allocation challenge can prove useful [4]. Max–min power control and equal power allocation are also two popular choices for power control [4,5] and will be analyzed in this work for a scalable FDD cell-free environment.

In essence, it is significant for a cell-free framework to be scalable with respect to the increase in the number of users joining the network [10,13]. The challenge of implementing a scalable FDD cell-free framework prevails, and therefore, it is imperative to provide a comparative analysis in FDD as well for efficient data sharing and processing even for a large number of users [5]. This work presents a scalability analysis of an FDD-based system in a cell-free network by utilizing angular reciprocity and angle-based precoding/combining and power control algorithms analyzed in this context. For an FDD-based cell-free framework to be scalable, there are two important conditions: (1) the propagation channel must be sufficiently sparse to benefit from angular reciprocity, a feature that is inherent in millimeter wave massive MIMO, and (2) the number of pilots should be independent of the number of UEs. The second condition is the focus of our current work, where we utilize a millimeter wave cell-free massive MIMO framework with FDD protocol.

### 1.1. Paper Contribution and Organization

The scalability of the power control algorithm is analyzed for FDD cell-free, where a comparative analysis of two power control algorithms, max–min power control and equal power allocation, is performed. The results are presented in terms of improved spectral efficiency for both uplink and downlink.

This work focuses on a scalable cell-free framework, where APs operate in FDD mode with angle reciprocity, in which no feedback is needed from UEs. The utilization of angular reciprocity aids in the acquisition of multipath components from the uplink pilots using combining schemes. The main contributions of this paper include the scalability analysis of the FDD cell-free framework, where a comparative analysis of angle-based channel estimation, precoding schemes, and power control algorithms is performed through numerical simulations. These contributions are listed in detail as follows:

- This paper addressed the scalability conditions linked with angle-based FDD systems and also identifies the relationship of the number of orthogonal pilots with UEs.
- A complete mathematical model for a scalable angle-based FDD cell-free system is presented while considering a dynamic and cooperative clustering technique.
- The proposed scalable angle-based FDD system is evaluated thoroughly for minimum mean squared error (MMSE) and matched filtering (MF) precoding schemes via simulations for both the uplink and downlink channels. The results demonstrate significant performance gains compared to conventional MMSE and MF schemes with respect to spectral efficiency and computational complexity.
- The scalability of the power control algorithm is also analyzed for MMSE and MF precoding schemes in an FDD cell-free framework, where a comparative analysis of two power control algorithms, that is, max–min power control, and equal power allocation, is provided. The results are presented in terms of improved spectral and energy efficiency for both the uplink and downlink channels.

The rest of the paper is organized as follows: Section 1 describes cell-free network for massive MIMO, Section 2 deals with scalability analysis of FDD-based cell-free massive MIMO, Section 3 is dedicated to modeling and analysis of spectral and energy efficiency, Section 4 presents derivation of power control algorithm for scalable cell-free, Section 5 provides results and discussion and Section 6 concludes the research work.

### 1.2. Related Work

Massive MIMO is inherently a scalable technology because a large number of UEs can be multiplexed in spatial domain [14]. The dimensions of antenna arrays utilized in massive MIMO can also be increased according to the increase in the number of served UEs [15]. However, it was observed through a literature review that there are large variations of SNR in massive MIMO for both macro and micro cellular systems [10,16]. On the other hand, cell-free massive MIMO systems combine the benefits of both cellular massive MIMO systems and ultra-dense networks, such as improvement of the network efficiency and data rates [14]. Moreover, cell-free massive MIMO systems avoid weaknesses, such as large SNR variations of massive MIMO systems and interference of ultra-dense networks [14,15].

**Figure 1:**
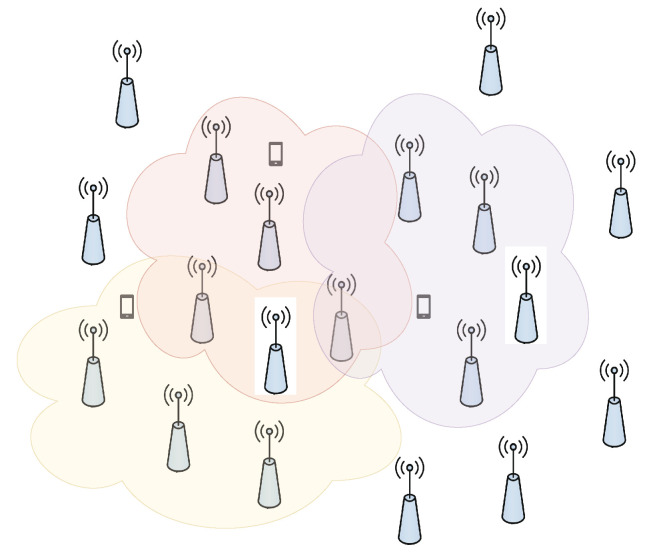
DCC framework for overlapping clustering scheme.

The cell-free framework has evolved from its original form of network MIMO or coordinated multipoint (CoMP) implementation [14]. In network MIMO, all the APs were assumed to have knowledge of CSI for the whole network and were supposed to transmit this knowledge to all the users. However, this form of cell-free, although theoretically preferred, was practically not possible, since there would be an immense increase in the signaling overhead for training and data transmission [16,17]. Authors in [18] suggested reducing the signaling overhead by sharing only the local AP’s CSI at the cost of the network’s ability to jointly mitigate the interference. However, interference mitigation in cooperative multicell networks presented by authors in [18], is better compared to non-cooperative cellular networks. The approach to local CSI sharing is to serve a single UE by only a subset of APs. Therefore, fronthaul signaling is performed for cooperation as well as data transmission at a local level, where each UE communicates with its neighboring APs only. There are two approaches to this kind of network operation, namely, network-centric and user-centric [19]. In the network-centric approach, APs are divided over the whole coverage area into non-contagious clusters. Inside each cluster, the UEs are served by the APs that provide the CSI estimates and payload data [20,21]. Although this technique is better than network MIMO, it provides only slight performance improvement with reference to interference mitigation [22]. An improved version of the network-centric approach, that is, the user-centric approach, is suggested by authors in [23,24], where the whole coverage area is divided into overlapping clusters of APs. This clustering is based on the ability of APs to serve the UEs with strong channel conditions, as shown in Figure 1 [23,24]. Moreover, Figure 1 suggests that different APs in a cluster serve different UEs, and these AP clusters adapt and change according to the variations in channel conditions or UE’s location due to their movement. A user-centric cooperation approach is suggested and is named “dynamic cooperation clustering (DCC)” by the authors of [25,26].

For the effective utilization of communication resources in massive MIMO systems, the choice of an appropriate duplexing scheme is vital [27,28]. The research in the area of cell-free massive MIMO is usually centered on TDD protocol, as cited by authors in [29,30] and its scalability is also analyzed in another cited research work [4]. An FDD-based cell-free massive MIMO system is considered in [31], with angle reciprocity, and the authors provide simulation results to confirm that the angle reciprocity-based FDD performs better compared to the subspace-based scheme. In addition, the cell-free framework provides significant improvement in energy efficiency for FDD-based systems [31].

The application of an accurate channel estimation technique for an FDD-based cell-free network is significant in the acquisition of reliable CSI estimates in the overall performance of the network. An FDD-based cell-free network is proposed by authors in [32,33] that utilizes a compressed sensing and deep learning-based estimation scheme for channel gains and simulation results confirm that the suggested estimation scheme provides reduced pilot overhead and improved system performance in terms of normalized MSE for the channel estimation. Authors in [5], suggested angular reciprocity-based precoding/beamforming schemes and provide spectral and energy efficiency expressions for an FDD cell-free network. The research work cited in [5] presents detailed numerical simulations for the mathematical model of angle-based FDD cell-free framework, however, the number of users is kept equal to the number of utilized orthogonal pilots. Therefore, the suggested system is not scalable for a large number of users because the number of orthogonal pilots is limited.

We have presented the comparison of the proposed scalable angle-based cell-free massive MIMO network and the relevant techniques with reference to literature and highlighted their major differences in Table 1. In view of this discussion, scalability analysis of FDD cell-free is needed, and therefore, in this work, we present scalability analysis of FDD cell-free with efficient precoding/combining schemes. The work in [4] details scalable TDD cell-free, however, the coverage of FDD scheme is better compared to TDD due to inherent interference mitigation, as uplink and downlink frequencies are distinct [5]. Therefore, the comparative analysis of the impact of the large number of UEs represented by *K* in the FDD cell-free framework is crucial for future wireless communication. In addition, for a large number of UEs connecting to the network, the number of APs can be optimized, which is required for an intended geographical area in a scalable FDD cell-free network [5]. Unlike [4], this work considers an FDD-based cell-free framework in the context of scalability, and suggests angle-based precoding for both minimum mean squared error (MMSE) and matched filtering (MF). Moreover, this work presents results based on a comparative analysis of MF and MMSE by considering multiple antennas per AP, since the FDD scheme leverages the antenna gains and provides an improvement in spectral efficiency.

The concept of angle-based estimation has been considered in subspace-based schemes [35,36], for cellular wireless networks including multiple-signal classification (MUSIC) algorithms and estimation of signal parameters by rotational in-variance technique (ESPRIT) [37]. Interested readers can read more on ESPRIT and MUSIC algorithm in [38,39,40]. However, these schemes are not scalable due to their higher computational cost and, therefore, are not suitable for cell-free massive MIMO frameworks. Authors in [41] presented a compressed sensing-based channel estimation scheme for an FDD cell-free network that achieves reduced pilot overhead compared to the MMSE scheme. This scheme gives improvement in spectral efficiency that is close to the conventional MMSE with orthogonal pilots, whereas, the energy efficiency of the proposed scheme by authors in [42] is better than the MMSE scheme. However, there is no scalability analysis presented for FDD cell-free framework in [42]. Authors in [43] suggest a Rician K-adaptive feedback technique with zero-forcing precoding and simulation results confirm an improved average rate for an FDD-based cell-free network. Considering the fact that none of the works in the literature review refer to the scalability of an FDD-based cell-free massive MIMO network, our proposed work presents a solution in this context.

## 2. Cell-Free Network for Massive MIMO

This work considers a frequency division duplex (FDD)-based cell-free massive MIMO setting with *K* single antenna users and *N* APs equipped with *M* antennas. The system model is derived from [4], whereas, the angle-based estimation techniques is deduced from [5]. All the APs are randomly distributed over the intended geographical area and are connected to a central processing unit (CPU). The UEs are distributed uniformly and independently in the entire coverage area [44]. Cell-free mMIMO network ensures joint coherent transmission and reception to the users over the whole coverage area. We assume that even for a large value of *K* such that $K\overset{}{\to }\infty ,N\geq K$ [4]. The cell-free network here is based on FDD protocol with angular reciprocity, where there is a pilot transmission phase for training and estimation purposes followed by a payload data transmission phase. The coherence time $\tau_{c}$ in the angular domain is much greater than its time domain counterpart [5,35]. We consider a fairly large $\tau_{c}$ such that $\tau_{c}$ is always greater than the number of UEs being served, and $\tau_{p}$ represents a number of pilot symbols that are mutually orthogonal. $\tau_{p}$ is assumed to be a constant, that is independent of *K* [45,46].

The channel model is assumed to be geometric with *L* propagation paths [32,47]. The FDD-based system has partial reciprocity in angle domain [5,45]. Therefore, we can assume that angles of arrival (AoAs) in the uplink and angles of departure in the downlink are almost equal. Similarly, the large-scale fading coefficients for both the up/downlink are similar. Since the FDD protocol shows partial reciprocity, small-scale fading coefficients vary with the uplink and downlink frequencies [5]. We denote the channel between *n*th AP and *k*th UE as $h_{kn}\in C^{M}$. Let P=MN represent the number of antennas in the whole coverage area. The realization of Rayleigh fading channel in a coherence block is given as $h_{kn}∼N\left(0,R_{kn}\right)$ such that $R_{kn}\in C^{M\times M}$ represents the spatial correlation matrix [4,5,32,47]. Considering this, the M×1 channel vector is represented as

(1) $$\begin{array}{c} h=\frac{1}{\sqrt{L}}\sum_{j=1}^{L}q\left(\phi_{j}\right)\beta_{j}\alpha_{j} \end{array}$$

where $q(\phi_{j})$ is the array steering vector, $\alpha_{j}$ represents the complex path gain, such that $\alpha_{j}∼CN\left(0,1\right)$ is the small scale fading, whereas $\beta_{j}$ represent large-scale fading coefficients including shadowing, path loss and spatial correlation for the *j*th path [4,5,44]. All the APs in the cell-free framework are independently distributed and, therefore, perform the estimation of multipath components in a distributed manner and channel vectors for *i*th AP with *j*th AP giving $E\{h_{ki}h_{kj}^{*}\}=0$∀i≠j. Therefore, the channel vector is represented in the matrix form as

(2) $$\begin{array}{c} H=\frac{1}{\sqrt{L}}QB\alpha \end{array}$$

where $Q_{M\times L}=\left[q\left(\phi_{1}\right),\ldots ,q\left(\phi_{L}\right)\right]$, $B_{L\times L}=diag\left(\beta_{1},\ldots ,\beta_{L}\right)$ and $\alpha_{L\times 1}={\left[\alpha_{1},\ldots ,\alpha_{L}\right]}^{T}.$

It is worth mentioning here that the small scale fading parameter given in α vector is frequency dependent whereas, the large-scale fading coefficients $\beta_{j}$ in B are independent of frequency for an angle coherence interval $\tau_{c}$. The system model in this work follows from [4,5,26,45] with partial reciprocity in the FDD protocol, where multipath components for both up/downlink are assumed to be independent and identically distributed (i.i.d.) random variables.

The wireless systems have evolved from conventional MIMO [48,49] to massive MIMO, and this evolution offers a great deal of benefits for future-generation wireless networks. In the following section, we build the FDD cell-free massive MIMO system model (more details can be found in [50,51]) and analyze its scalability for a large number of UEs *K* joining the network such that $K\overset{}{\to }\infty$.

## 3. Scalability Analysis for FDD-Based Cell-Free Massive MIMO

A cell-free massive MIMO framework is deemed scalable if an increase in the number of users such that $K\overset{}{\to }\infty$ does not impact the implementation of the following [4]:

- computation of estimates of the wireless channel for *K* UEs.
- combining/beamforming computation for up/downlink.
- control signaling for fronthaul (data and feedback).
- optimization of power control.

In a scalable system, the complexity of implementation and requirement for resources must remain finite for all APs, even with a large number of UEs joining the network [4,8]. In FDD-based systems with angular reciprocity, an increase in the number of UEs such that $K\overset{}{\to }\infty$ requires an equal increase in system resources such as the number of *N* Aps and *M* antenna elements per AP. Therefore, the dependence of implementation complexity shifts from *K* to the product MN, which is much larger than *K*. However, the solution is the localized cell-free framework, where APs are equipped with their own processors and connection with the fronthaul. The APs only exchange local CSI estimates among each other, and network-wide CSI exchange is not utilized in cell-free networks [5]. In addition, our FDD-based cell-free framework can leverage from the dynamic cooperation clustering (DCC) scheme that allows APs to communicate with only a subset of UEs $A_{n}$ instead of all *K* UEs [4,25,26]. To check the scalability of our FDD-based cell-free framework, we further assume that set $A_{n}$ comprises of UEs served by at least one antenna of the *n*th AP, as,

(3) $$\begin{array}{c} A_{n}=\left\{k:tr(D_{nk})\geq 1,k\in 1,2,\ldots ,K\right\}. \end{array}$$

Let $D_{kn}\epsilon C^{N\times N}$ be defined as a set of matrices, each a diagonal one, to represent the communication from *n*th AP to *k*th UE. The corresponding diagonal element of $D_{kn}$ is asserted to 1 if AP *n* wishes to transmit to UE *k*, and is 0 otherwise, i.e.,

(4) $$\begin{array}{c} D_{kn}=\{\begin{array}{cc} I_{M} & k\in A_{n}\\ 0_{M} & k\notin A_{n}. \end{array} \end{array}$$

### 3.1. Uplink Pilot Training and Channel Estimation

As explained in Section 1, $\tau_{c}$ represents the angular coherence time such that $\tau_{c}=\tau_{p}+\tau_{d}$, with $\tau_{p}$ pilot symbols and $\tau_{d}$ data symbols. Each pilot sequence comprises pilot length $\tau_{p}$ and has a unit norm. In an FDD cell-free network with angular reciprocity, the value of $\tau_{c}$ is comparatively large, thus, pilot sequences are assumed as mutually orthogonal. When UEs join the coverage area of our cell-free network, they are assigned pilots according to the algorithm suggested in [4]. It is worth mentioning here that the estimation of multipath components is performed by each AP independently by utilizing the pilot sequences. The *n*th AP $\in M_{k}\subset \left\{1,2,\ldots ,N\right\}$ is required to perform multipath component estimation locally, for its neighboring $A_{n}$ UEs such that $A_{n}\subset \left\{1,2,\ldots ,K\right\}$. There is no cooperation or sharing of information among the APs for the estimation of multipath components. We assume $\tau_{p}$ pilot symbols, utilizing (2), the received signal $Y_{n}$∈$C^{M\times \tau_{p}}$ at the *n*th AP, for FDD protocol, is presented in matrix form as [5]

(5) $$\begin{array}{c} Y_{nk}=\sqrt{\frac{\rho }{L}}Q_{nk}B_{nk}\alpha_{nk}\tau_{p}+U_{nk} \end{array}$$

where ρ is the transmit power for *k*th UE and $U_{nk}∼\left(0,\sigma_{n}^{2}\right)$ is the AWGN matrix such that $U_{nk}$∈$C^{M\times \tau_{c}}$.

The estimation of the FDD-based cell-free massive MIMO framework is performed by first utilizing the DFT-based angle of arrival (AoA) estimation after acquiring the multipath components estimates directly via uplink training and then applying this angle information to estimate the large-scale fading coefficients. The overall complexity of angle-based estimation is reduced when beamforming/combining schemes are applied that is based on the estimation of both of these components. The AoA estimation in [5] has better accuracy due to the utilization of the angle rotation technique, whereby, a phase shift is introduced into the initial estimates, resulting in accurate peak elements. Therefore, the estimated AoA matrix is given as

(6) $$\begin{array}{c} {\hat{Q}}_{nk}=\left[q\left({\hat{\phi }}_{1,nk}\right),q\left({\hat{\phi }}_{2,nk}\right),\ldots ,q\left({\hat{\phi }}_{L,nk}\right)\right] \end{array}$$

Next, these AoA estimates ${\hat{Q}}_{nk}$ are used for computing large-scale fading coefficients $\beta_{nk}$. The covariance matrix ${\hat{R}}_{nk,z}$ is related to $\beta_{L,nk}$ and, therefore, large-scale fading coefficients can be obtained, as suggested in [5],

(7) $$\begin{array}{c} {\hat{\beta }}_{nk}=diag\left(\beta_{1,nk},\beta_{2,nk},\ldots ,\beta_{L,nk}\right)={\hat{R}}_{nk,z} \end{array}$$

The estimation algorithm is run over the search grid V for all paths *L* and all antennas *M* that incorporates the complexity of DFT operation as well as angle rotation procedure, $\Delta \phi_{l}\in \left[-\frac{\pi }{M},\frac{\pi }{M}\right]$, where $\Delta \phi_{l}$ is defined as the angle rotation parameter [5,41]. The purpose of this grid parameter V is to establish the accuracy and computational complexity of the angle-based estimation algorithm [5]. Table 2 shows the comparison of the complexity of estimation for angle-based, MUSIC, and ESPRIT algorithms; $U_{i}$ is the search grid parameter for ESPRIT algorithm, such that, $U_{i}>>V_{i}$.

It is worth mentioning that the performance of the angle-based FDD estimation degrades slightly for a large grid size V. It is also evident that the estimated values of $\hat{\beta }$ and $\hat{Q}$ remain fixed for the coherence interval and, therefore, only estimated once during the entire communication. Authors in [4] have suggested pre-computing the channel statistics at *n*th AP and performing the channel estimation for each UE based on that pre-computed statistics matrix, thus requiring fewer complex multiplications.

### 3.2. Data Transmission Phase for Downlink

For the downlink data transmission to *K* users, APs use precoding vector ${\hat{w}}_{nk}$ of length M×1. There is no exchange of information among APs regarding the precoding vectors. The downlink signal of *n*th AP to *k*th UE, $x_{n}$ is represented as

(8) $$\begin{array}{c} x_{n}=\rho^{d}\sum_{k=1}^{K}{\hat{w}}_{nk}s_{k}^{d} \end{array}$$

where $s_{k}^{d}$ represents the data symbol and $\rho^{d}$ depicts the maximum transmit power for *k*th user.

### 3.3. Angle Based Beamforming

The precoding/beamforming is performed to suppress the noise and interference in an efficient manner. The angle-based precoding vector for matched filtering is given as

(9) $$\begin{array}{c} {\hat{w}}_{nk}=\frac{{\hat{G}}_{nk}}{\left|\right|{\hat{G}}_{nk}\left|\right|}\Upsilon_{nk} \end{array}$$

where $\Upsilon_{nk}$ represents the complex normalized weight of the *k*-th user, such that $E[|\Upsilon_{nk}{|}^{2}]=1$ where $\Upsilon_{nk}={\left[\Upsilon_{nk,1},\ldots ,\Upsilon_{nk,L}\right]}^{T}$ and ${\hat{G}}_{nk}={\left[{\hat{g}}_{nk,1},{\hat{g}}_{nk,2},\ldots ,{\hat{g}}_{nk,L}\right]}^{T}$.

Matched filtering is a simple precoding technique that only requires estimates of AoA and large-scale fading coefficients for the channel between *n*th AP and *k*th UE. ${\hat{G}}_{nk}$ is the angle-based precoding matrix, and for matched filtering, it can be represented as

(10) $$\begin{array}{c} {\hat{G}}_{nk}^{mf}={\hat{Q}}_{nk}{\hat{B}}_{nk} \end{array}$$

Finally, angle-based MMSE, given as ${\hat{G}}_{nk}^{a-mmse}$, provides precoding with efficient performance against interference and noise suppression as well as estimation error. ${\hat{G}}_{nk}^{a-mmse}$ is given as

(11) $$\begin{array}{c} {\hat{G}}_{nk}^{a-mmse}={\left(\sum_{k=1}^{K}\left({\hat{Q}}_{nk}{\hat{B}}_{nk}{\hat{Q}}_{nk}^{H}{\hat{B}}_{nk}^{H}+{ı}_{nk}\right)+\sigma_{m}^{2}I_{M}\right)}^{-1}\times {\hat{Q}}_{nk}{\hat{B}}_{nk} \end{array}$$

where ${ı}_{nk}$ depends on the variance of difference among up/downlink multipath components ($\upsilon_{n}\;\text{and}\;\beta_{l}$) [5].

For the downlink, we utilized DCC and modified the received signal at *k*th UE as

(12) $$\begin{array}{c} y_{k}=\sum_{n=1}^{N}h_{kn}^{H}\sum_{k=1}^{K}D_{nk}{\hat{w}}_{nk}\rho^{d}+U_{n}=h_{k}^{H}\sum_{k=1}^{K}D_{k}{\hat{w}}_{k}\rho^{d}+U_{n} \end{array}$$

where $U_{n}∼N_{C}\left(0,\sigma^{2}\right)$ is the noise at the receiver. It is apparent from Equation (12) that *n*th AP will transmit to *k*th UE if $D_{nk}{\hat{w}}_{nk}={\hat{w}}_{nk}$ or when $D_{nk}\neq 0$.

We argue that if the number of users *K* increases such that $K\overset{}{\to }\infty$, but the cardinality $|A_{n}|$ remains finite for all n=1,2,…,N then the FDD-based cell-free network is scalable (proof follows from [4]). We utilized the algorithm suggested by [4] for the tasks such as initial joint access, clustering, and pilot allocation. Based on the assumption that every AP serves a maximum of one UE for one pilot sequence, and all *M* antennas of that AP will be utilized to serve all those UEs, Equation (4) describes the communication among *n*th AP and *k*th UE.

It is mentioned in Section 2 that $\tau_{p}$ is assumed to be a constant and independent of *K*, an increase in *K* does not imply a similar effect on the value of $\tau_{p}$[35,36]. This, combined with the argument of $A_{n}$ having constant cardinality, provides sufficient conditions for the scalability of an FDD-based cell-free framework. The algorithm for clustering, joint initial access, and pilot allocation is scalable because the complexity of the tasks of estimation and precoding is independent of *K* and only depends on $k\in A_{n}$ that is a maximum of one pilot per UE.

### 3.4. Data Transmission Phase for Uplink

All *K* UEs send payload in the uplink at the same time. The data symbols $s_{k}^{u}$, such that $E[|s_{k}^{u}{|}^{2}]=1$, are sent towards the APs and received signal is represented as

(13) $$\begin{array}{c} y_{n}^{u}=\rho^{u}\sum_{k=1}^{K}h_{nk}s_{k}^{u}+U_{n}^{u} \end{array}$$

where $\rho^{u}$ is the transmit power in the uplink and $n_{n}^{u}$ represents the noise added at *n*th AP, such that $n_{n}^{u}∼CN\left(0,\sigma_{n}^{2}\right)$. At *n*th AP, the signal is multiplied by the M×1 combining vector $v_{nk}$. Finally, the signal is sent for detection to the CPU via the fronthaul link. The received signal at the CPU is expressed as

(14) $$r_{k}^{u}=\sum_{k=1}^{K}\sum_{n=1}^{N}\rho^{u}v_{nk}^{H}h_{nk}s_{k}^{u}+\sum_{n=1}^{N}v_{nk}^{H}U_{n}^{u}$$

The original cell-free concept employs network-wide decoding of the uplink, where *n*-th AP chooses the combining vector $v_{nk}\in C^{N}$ for *k*-th UE and performs local computations. For a cell-free network with DCC, the APs acquire the CSI estimates locally during pilot transmission. Although all APs will receive the signal from all *K* UEs, yet, only a subset of these APs perform signal detection, therefore, the estimated ${\hat{R}}_{k}=\sum_{n=1}^{N}v_{nk}^{H}D_{kn}y_{n}^{u}$ is modified as

(15) $$\begin{array}{c} {\hat{R}}_{k}=v_{k}^{H}D_{k}h_{k}s_{k}^{u}+\sum_{j=1,j\neq k}^{K}v_{k}^{H}D_{k}h_{j}s_{j}^{u}+v_{k}^{H}D_{k}U_{n}^{u} \end{array}$$

where $v_{k}={\left[v_{k,1}^{T},v_{k,2}^{T},\ldots ,v_{k,N}^{T}\right]}^{T}\in C^{N}$ represents the overall combining vector for all APs and $U_{n}$ represents the combination of all noise vectors, $U_{n}={\left[U_{1}^{T},\ldots ,U_{N}^{T}\right]}^{T}\in C^{N}$. given that, $D_{k}=diag\left(D_{k1},\ldots ,D_{kN}\right)\in C^{N\times N}$ represents a block diagonal matrix, and only the APs for which $D_{jn}\neq 0_{M}$ will apply a combining vector at the received signal. Two cooperation mechanisms are available for APs: centralized combining and distributed combining. In centralized combining-based cooperation, APs send the pilot and data signals to the CPU via a fronthaul link. The CPU performs channel estimation and signal detection in a centralized manner. For every coherence time, *n*th AP is required to send approximately $\tau_{p}M$ complex values for the pilot as well as $\tau_{p}^{u}M$ complex values for the received signal. In distributed combining, *n*-th AP chooses vector $v_{kn}$ based on local channel estimates $\forall [{\hat{h}}_{jn}:j\in A_{n}]$ and performs the computation of local estimates as ${\hat{R}}_{kn}=v_{kn}^{H}y_{n}^{u}$. The *n*th AP is required to compute only the local estimates of $|A_{n}|$ users and $\tau_{p}^{u}\left|A_{n}\right|$ complex values are sent every coherence interval to CPU, which are independent of *K* and therefore, make the combining scalable. It may be noted that for distributed combining cases, the CPU has no knowledge of channel estimates, making it unlikely to obtain the expression for spectral efficiency in the uplink. A popular solution is “use-and-then-forget bound” in cell-free massive MIMO systems (details are given in [4,44]), for a choice of combining vector $v_{kn}$, such that $D_{j}=I_{N}\forall j$. We utilized this approach for the signal received, given by Equation (15) and angle-based combining as represented in the following subsection.

### 3.5. Angle-Based Combining

The received signal combining vector of *n*th AP and *k*th UE is given as [5]

(16) $$\begin{array}{c} v_{nk}=\sum_{j=1}^{L}\gamma_{nk,j}{\hat{c}}_{nk,j}={\hat{C}}_{nk}\gamma_{nk} \end{array}$$

where ${\hat{C}}_{nk}=\left[{\hat{c}}_{nk,1},\ldots ,{\hat{c}}_{nk,L}\right]$, $\gamma_{nk,j}=\frac{1}{L}$ and $\gamma_{nk}=\left[\gamma_{nk,1},\ldots ,\gamma_{nk,L}\right]$.

We utilized the uplink–downlink duality to obtain the combining vector expressions for angle-based matched filtering and angle-based MMSE combining as presented in (10) and (11). This duality implies that ${\hat{C}}_{nk}={\hat{G}}_{nk}$.

## 4. Analysis of Spectral and Energy Efficiency

Based on the beamforming and combining results obtained so far, we analyze the performance of this scalable cell-free massive MIMO framework for per UE spectral and energy efficiency in this section.

### 4.1. Spectral Efficiency of FDD-Based Cell-Free

The per-user spectral efficiency in the downlink is computed by utilizing the precoding scheme and is represented as [5]

(17) $$\begin{array}{c} SE_{k}^{d}=log_{2}\left[1+SINR_{k}^{d}\right]≃log_{2}\left(1+\frac{\rho^{d}R_{k}^{d}}{\rho^{d}I_{jk}^{d}+\rho^{d}F_{k}^{d}+\sigma_{dl}^{2}}\right) \end{array}$$

where $R_{k}^{d}$ denotes the desired signal strength for cell-free framework, $I_{jk}^{d}$ is the interference term due to *j*-th UE and $F_{k}^{d}$ is the uncertainty in the beamforming gain defined as zero-mean random variable [5].

Therefore, for the proposed, scalable cell-free framework, $SINR_{k}^{d}$ is expressed as

(18) $$\begin{array}{c} SINR_{k}^{d}=log_{2}\left(\frac{\rho^{d}E[||{\hat{h}}_{k}^{H}D_{k}{\hat{w}}_{k}{\left|\right|}^{2}]}{\rho_{j}^{d}E[||h_{k}^{H}D_{j}{\hat{w}}_{j}{\left|\right|}^{2}]+\rho^{d}E[||{\tilde{h}}_{k}^{H}D_{k}{\hat{w}}_{k}{\left|\right|}^{2}]+\sigma_{dl}^{2}}\right) \end{array}$$

The per-user spectral efficiency for combining schemes in the uplink can be deduced from the downlink case by substituting ${\hat{w}}_{j}$ with $v_{j}$ and is presented as

(19) $$\begin{array}{c} SE_{k}^{u}≃log_{2}\left(1+\frac{\rho^{u}E[||{\hat{h}}_{k}^{H}D_{k}v_{k}{\left|\right|}^{2}]}{\rho_{j}^{u}E[||h_{k}^{H}D_{j}v_{j}{\left|\right|}^{2}]+\rho^{u}E[||{\tilde{h}}_{k}^{H}D_{k}v_{k}{\left|\right|}^{2}]+\sigma_{dl}^{2}\left|\right|v_{k}{\left|\right|}^{2}}\right). \end{array}$$

### 4.2. Scalability Analysis of Spectral Efficiency for Uplink

Considering the SINR expressions for the uplink combining scheme with $v_{nk}$, where $k\in D_{nk}$, the *n*-th AP is required to choose the combining vector based on the ${\hat{h}}_{jn}={\hat{Q}}_{jn}{\hat{B}}_{jn}$ such that $\left\{{\hat{h}}_{jn}:j\in A_{n}\right\}$ with no knowledge of CSI estimates at other APs. Any of the combining schemes mentioned before (angle-based MF and angle-based MMSE) can be utilized to compute the SINR for spectral efficiency. The simplest is the angle-based matched filtering combining scheme. Angle-based MMSE combining gives performance improvement; however, it is not a scalable option. A local partial MMSE combining scheme is suggested in [4] that is inspired by the partial MMSE (P-MMSE) scheme [27] and is given as

(20) $$\begin{array}{c} v_{nk}^{lp-mmse}=\left\{\sum_{j\in A}{\left(\left({\hat{h}}_{jn}{\hat{h}}_{jn}^{H}+{ı}_{jn}\right)+\sigma_{ul}^{2}I_{M}\right)}^{-1}{\hat{h}}_{jn}\right\} \end{array}$$

where ${ı}_{jn}$ is the collective estimation error between the variances of the uplink and downlink multipath components [5]. The difference in Equations (11) and (20) is that LP-MMSE in (20) is only considering the estimates of UEs that are served by *j* APs $\left(\forall j\in A_{n}\right)$, since, $|A_{n}|<\tau_{p}$ and $\tau_{p}$ is assumed independent of *K*, the LP-MMSE becomes a scalable scheme even for $K\overset{}{\to }\infty$. The complexity of LP-MMSE is lower compared to the centralized P-MMSE [4]. In addition, the computational order of LP-MMSE remains finite even for $K\overset{}{\to }\infty$ due to the finite number of multiplications required. The spectral efficiency of LP-MMSE is obtained easily through Monte Carlo simulations.

### 4.3. Scalability Analysis of Spectral Efficiency for Downlink

The spectral efficiency of downlink can be obtained from the uplink–downlink duality by considering the uplink combining vector given as $\left[v_{j}:j=1,2,\ldots ,K\right]$ with the power of each UE as $\left[\rho_{i}^{u}:i=1,2,\ldots ,K\right]$. We used the uplink–downlink duality by first expressing beamforming per UE ${\bar{w}}_{j}$ as

(21) $$\begin{array}{c} {\bar{w}}_{j}=\frac{v_{j}}{E[v_{j}^{H}D_{j}v_{j}]} \end{array}$$

by utilizing an appropriate power control algorithm in the downlink, where

(22) $$\begin{array}{c} \sum_{j=1}^{K}\frac{\rho_{j}}{\sigma_{n,dl}^{2}}=\sum_{j=1}^{K}\frac{\rho_{j}}{\sigma_{n,ul}^{2}} \end{array}$$

such that for *k*-th UE, $SINR^{d}=SINR^{u}$ for all *k*.

The two cooperation mechanisms for precoding are centralized precoding and distributed precoding. In centralized precoding, estimates for downlink are obtained by using the uplink–downlink duality. Once the estimates are computed, we deduce the precoding matrix from Equation (21), and next, CPU constructs the downlink signal,

(23) $$\begin{array}{c} x_{n}^{d}=\sum_{j=1}^{K}\rho_{j}D_{jn}{\bar{w}}_{jn}\zeta_{j} \end{array}$$

and sends it to the AP through fronthaul, where $\zeta_{j}$ corresponds to the data signal for downlink. In every coherence interval, *n*th AP has to send $\tau_{p}M$ complex values to the CPU and receive $\tau_{p}M$ values from the CPU.

In distributed precoding, *n*th AP chooses the precoding vector ${\bar{w}}_{j}$ by using only the local channel estimates $\left[{\hat{h}}_{jn}:j\in A_{n}\right]$ for scalable cell-free framework. Therefore, in every coherence block, $\tau_{d}A_{n}$ complex values are exchanged among *n*th AP and CPU. This work utilizes distributed precoding mechanism. It is worth mentioning here that only angle-based matched filtering, and angle-based LP-MMSE precoding schemes are scalable, such as in the case of combining schemes.

### 4.4. Overhead of Scalable Angle Based Scheme in FDD Cell-Free

Table 3 provides the comparison of angle-based MMSE (non-scalable) with scalable angle-based LP-MMSE and MF schemes. The total order of complexity for original angle-based MMSE is the sum of DFT operations performed for multipath components estimation MlogM, order of angle rotation performed over the search grid VML and number of complex scalers transmitted to CPU for decoding of signal in each coherence block, which are $M\tau_{p}$ for centralized scheme and $|A_{n}|\tau_{u}$ for the distributed case. This complexity increases linearly with an increase in the number of users joining the network. It is evident from Table 3 that angle-based MMSE without utilizing DCC is not scalable for large *K* due to its linear dependence on the number of UEs. The LP-MMSE scheme, however, is scalable due to its dependence on $M_{k}$ instead of *K* because $M_{k}\subset K$ and does not scale linearly with *K*.

### 4.5. Energy Efficiency

Energy efficiency is a good measure for performance evaluation of FDD-based systems and is defined as the ratio of throughput in bits/s to the total power consumed in Watts. It is measured in bit/joule. It is given as

(24) $$\begin{array}{c} \eta =\frac{E\sum_{k=1}^{K}⋋SE_{k}^{u}}{P_{t}} \end{array}$$

where $⋋=\left(1-\frac{\tau_{p}}{\tau_{c}}\right)$. The total power consumed is the sum of power consumption at AP and the power utilized over the backhaul and control signaling [4,41]. As mentioned already, we assume $\tau_{p}$ to be independent of *K*. The power consumption of *n*th AP depends on the angle-based beamforming vector and the number of antennas *M*. Therefore, an increase in the number of UEs *K* will not affect the energy efficiency.

## 5. Power Control Algorithm for Scalable Cell-Free

Some works in the literature suggest performing power control at the CPU for scalable cell-free massive MIMO [4,5]. For angle-based schemes, coherence time is greater, therefore, low signaling overhead is incurred. Authors in [5] utilized a user-centric AP selection approach for power control that results in increased energy efficiency. Work in [4] proposed a trial-and-error approach toward power control. In this work, a network-wide power allocation was studied for centralized as well as distributed beamforming cooperation. The two approaches help APs to ensure conformity to the power constraint by serving a maximum number of users equal to the number of pilots with equal power per user. For equal power allocation with centralized precoding in the uplink, the power allocated to an *i*th UE is $\rho_{i}=\frac{\rho_{n}}{\tau_{p}}$, where $\rho_{n}$ is power of *n*th AP in the network [4]. We assume that in the uplink, all users transmit with maximum power *P* to enhance the spectral efficiency for all UEs, strong or weak, such that, $p_{i}=P,\;\mathrm{for}\;i=1,2,\ldots ,K$.

In this research, we also analyzed the performance of distributed approach for power control in the FDD cell-free network. The aim was to ensure that every user would be served by at least one AP and, therefore, would have non-zero spectral efficiency. The power control task in distributed scenarios is more complicated. In the distributed scenario, AP has to assign power to all the UEs being served by it. The two most common approaches for power control are equal power allocation and max–min power control algorithms [4]. Out of these two, the max–min power/weight control approach works very well in FDD cell-free networks, even if some users are far away from the serving AP [5]. For the users that are located far away from AP, the joint initial access algorithm ensures that such a user is served by at least one AP, which will allocate the UE a certain amount of transmission power, and thus, its spectral efficiency will also be non-zero. The max–min power control algorithm is discussed in the following subsection for FDD cell-free massive MIMO network.

### Max–Min Power Allocation for Downlink

After the large-scale fading coefficients and array angle steering vectors have been obtained, the coefficients $\Upsilon_{nk}$ for *n*th AP to *k*th UE are utilized for max–min power control [5],

(25) $$\begin{array}{c} \begin{array}{ccc} max & min & SE_{k}^{d}\\ \left\{\Upsilon_{nk,j}\right\} & k=1,\ldots ,K & \\ \text{given}\;\text{that}, & \sum_{k=1}^{K}\frac{{\hat{G}}_{nk}}{\left|\right|{\hat{G}}_{nk}\left|\right|}\Upsilon_{nk}\leq 1, & \forall m=1,\ldots ,M \end{array} \end{array}$$

where $SE_{k}^{d}$ is the downlink spectral efficiency for *k*th UE, and $\Upsilon_{nk}\geq 0\;\forall n,k\;and\;j,$ i.e., the algorithm will ensure maximization of the minimum data rate for UEs in the downlink. The best case scenario is that all UEs will achieve the same maximum data rate, and in the worst case scenario, UEs with weak channel conditions will be allocated lower power compared to the UEs with stronger channel link (based on $\Upsilon_{nk}$). The original form of this algorithm is not scalable for a large number of UEs in a cell-free network. However, for a cell-free network with scalable precoding/combining, the power distribution among the APs in the distributed precoding-based system is determined by the unit norm precoding vector $\bar{w_{j}}$. The max–min power control algorithm is utilized to compute ${\bar{w}}_{j}$, based on the $\Upsilon_{nk}$ coefficients. All the APs ensure adhering to the power constraint by serving a maximum of $\tau_{p}$ users and a maximum of $\frac{\rho_{n}}{\tau_{p}}$ power per user is allocated. Therefore, the maximum power control algorithm is scalable because the number of variables to be optimized is now independent of *K*.

## 6. Simulation Results

We provide numerical results for an FDD-based cell-free massive MIMO network and compare the performance of scalable angle-based precoding and combining schemes in terms of their spectral and energy efficiencies.

### 6.1. Simulation Setup

In this work, we used Monte Carlo simulations in MATLAB for an FDD cell-free network. As the literature suggests that FDD cell-free networks require fewer APs to provide the same coverage area compared to TDD-based cell-free networks, the interference in an FDD-based network will be inherently limited due to separation in the up/downlink frequencies [5]. In addition, the higher number of antennas per AP achieves spatial diversity gain for an angle-based FDD scheme. Therefore, FDD was expected to provide improved performance for the centralized scenario (N=100, M=8) compared to the performance in the case of the distributed scenario (N=200, M=4). We considered an area of $2\times 2\;km^{2}$ and a wrap-around scenario was assumed to mimic a very large network with randomly distributed APs [4] and 200 antennas per km${}^{2}$. The number of UEs was considered to be K=100 with uniform and independent distribution over the coverage area. Two scenarios were simulated: (i) N=100 and M=8, (ii) N=200 and M=4, therefore, the total number of serving antennas in the network remained the same, i.e., NM=800.

Initially, $\tau_{p}$ UEs connected with the FDD-based cell-free massive MIMO network. UEs were assigned random pilots from a set of orthogonal pilot sequences. We utilized the channel model as presented in [5], where APs were placed 10 m above the UEs such that a minimum distance between *n*th AP and *k*th UE was maintained. The rest of the simulation parameters are listed in Table 4.

### 6.2. Results and Discussion

**Figure 2:**
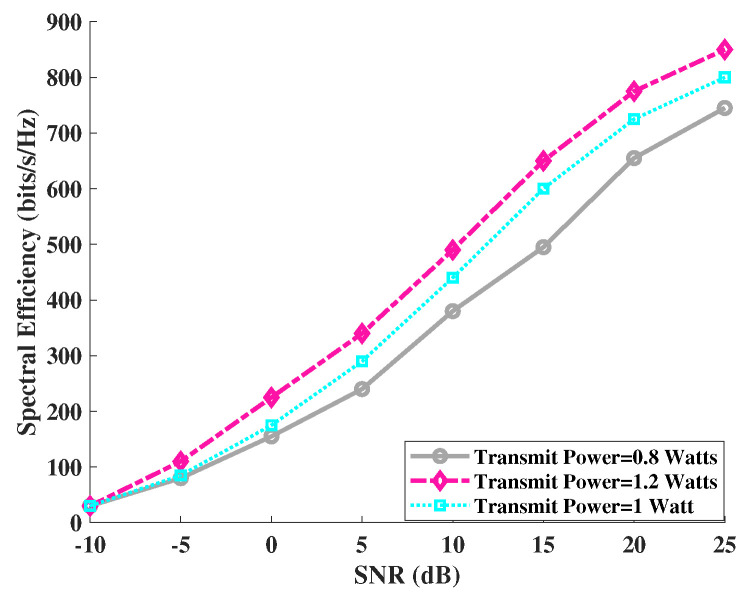
Comparison of spectral efficiency for angle-based LP-MMSE with increase/decrease in transmit power.

The transmit power of an AP is a major factor that can help evaluate the performance of a massive MIMO system in terms of energy and spectral efficiency. Therefore, we analyzed the impact of transmit power variations on the spectral efficiency of our proposed LP-MMSE scheme. Simulations were performed for the centralized scenario (N=100, M=8) with the following set of values for transmit power, [0.8 1 1.2] Watts. It is evident from Figure 2 that the increase in the downlink transmit power results in an increase in spectral efficiency, and the SNR vs. spectral efficiency graph shifts upwards. Similarly, a decrease in transmit power decreases the spectral efficiency and causes the graph of spectral efficiency to shift downwards. The trend shown in the graph is in line with the findings in the literature suggested by authors in [5]. Therefore, based on the result shown in Figure 2, we selected the value of 1 Watt for transmit power in subsequent simulations, since it represents an optimal value for improvement in both spectral and energy efficiency of the system.

**Figure 3:**
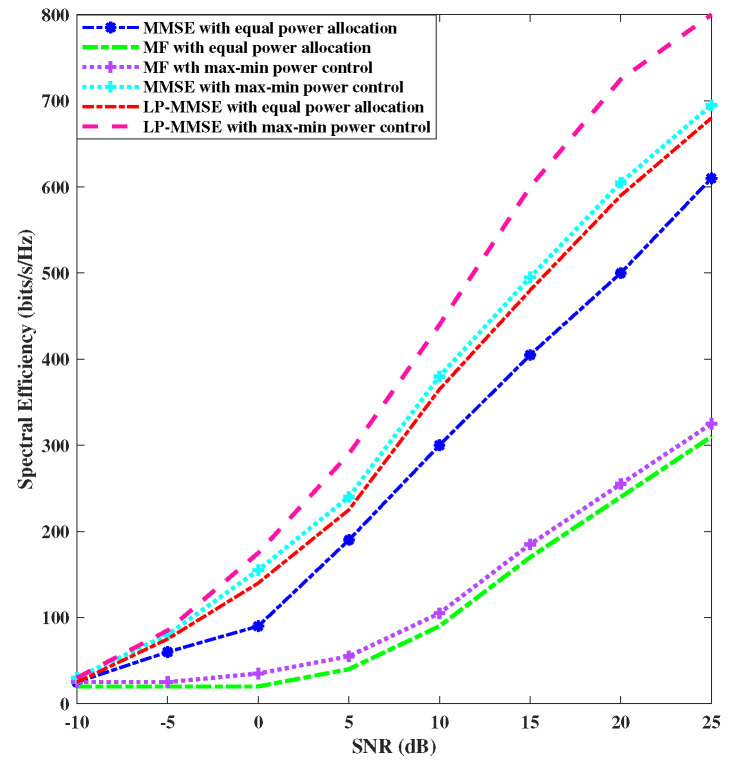
Comparison of downlink power control for angle-based MF, LP-MMSE, and MMSE schemes.

**Figure 4:**
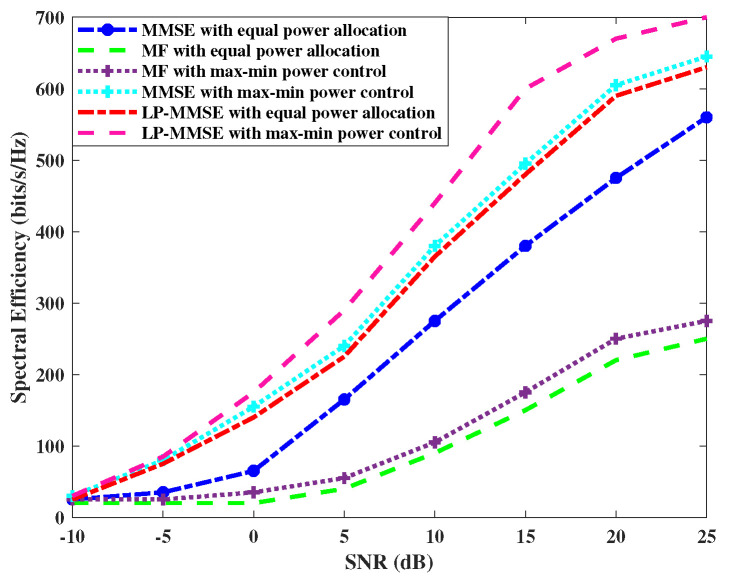
Comparison of uplink power control for angle-based MF, LP-MMSE, and MMSE schemes.

We analyzed the spectral efficiency performance of the max–min power control algorithm in both the uplink and downlink. Figure 3 shows the performance of MMSE, LP-MMSE, and MF schemes on the basis of spectral efficiency in the downlink by utilizing max–min power control versus equal power allocation. We considered the centralized scenario (N=100, M=8) and K=100 for our simulations. The results of the downlink case are shown in Figure 3, which depict that the spectral efficiency of LP-MMSE with max–min power control at 24 dBs is 800 bits/s/Hz, whereas, with equal power allocation, it is at 650 bits/s/Hz. Figure 4 compares the performance of the spectral efficiency in the uplink for the proposed scalable LP-MMSE, MMSE, and scalable MF schemes by utilizing max–min power control versus equal power allocation. Figure 4 shows that the spectral efficiency of LP-MMSE with max–min power control at 24 dBs is 690 bits/s/Hz, whereas, with equal power allocation, it is at 625 bits/s/Hz. Therefore, LP-MMSE with max–min power allocation reports a 10.4% increase in spectral efficiency over equal power allocation. It is apparent from these results that the max–min power control algorithm provides better spectral efficiency compared with equal power allocation in both the uplink and downlink. Moreover, the LP-MMSE with max–min power control provides significant performance improvement in terms of spectral efficiency over angle-based MF and MMSE schemes with max–min power control.

**Figure 5:**
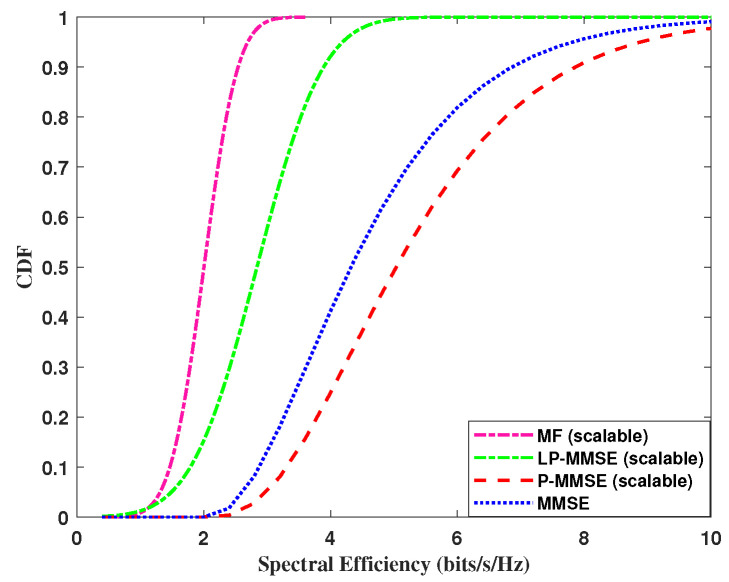
CDF of spectral efficiency for uplink, 100 APs and *M* = 8 antennas/AP.

**Figure 6:**
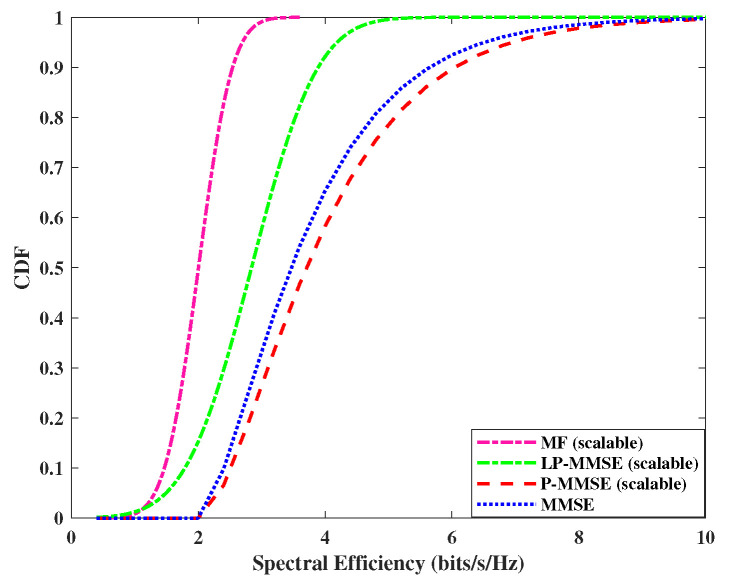
CDF of spectral efficiency for uplink, *N* = 200 APs and *M* = 4 antennas/AP.

Next, we analyzed the performance of the uplink per user spectral efficiency, using the expression in Equation (19). The cumulative distribution function (CDF) of DCC with angle-based precoding schemes including MF, MMSE, and scalable LP-MMSE are compared for the two scenarios and are shown in Figure 5 and Figure 6. In our case, the angle-based MF scheme shows improved performance over simple MF; however, its performance is still not on par with the angle-based LP-MMSE scheme. It is evident from Figure 5, that the scalable LP-MMSE outperforms both MF and MMSE schemes. The LP-MMSE scheme gives a significant improvement in spectral efficiency for 97% of the UEs, and its performance for the remaining 3% weak-channel condition users is still better compared to its MF counterpart. In addition, it is also shown in Figure 5 that FDD-based cell-free provides improved spectral efficiency in the scenario where the number of antennas per AP is higher, N=100, M=8 compared to the N=200, M=4 case in Figure 6, because of the increase in spatial diversity gains with angle-based beamforming. The gap in the performances of MF vs. LP-MMSE and MMSE vs. LP-MMSE is significant, where the latter is even greater, which endorses that our proposed scalable LP-MMSE scheme is the better choice for FDD cell-free network. In Figure 6, this performance gap reduces for N=200, M=4 with the decrease in number of antennas per AP.

**Figure 7:**
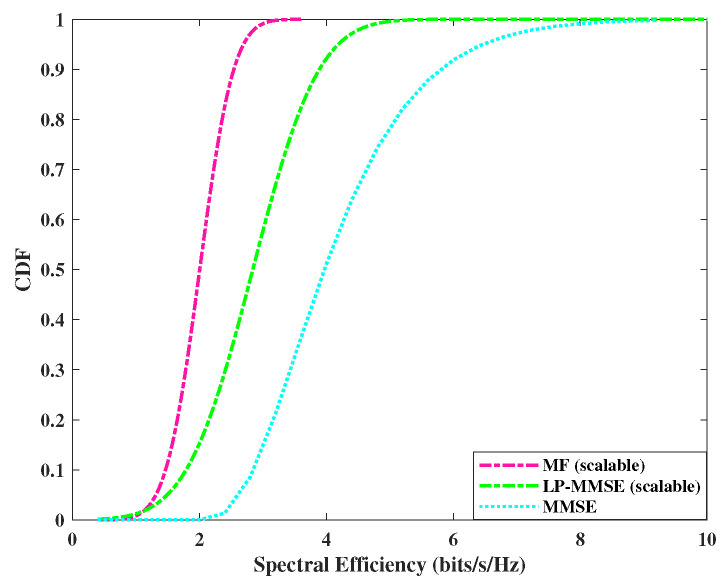
CDF of spectral efficiency for downlink, *N* = 100 APs and *M* = 8 antennas/AP.

**Figure 8:**
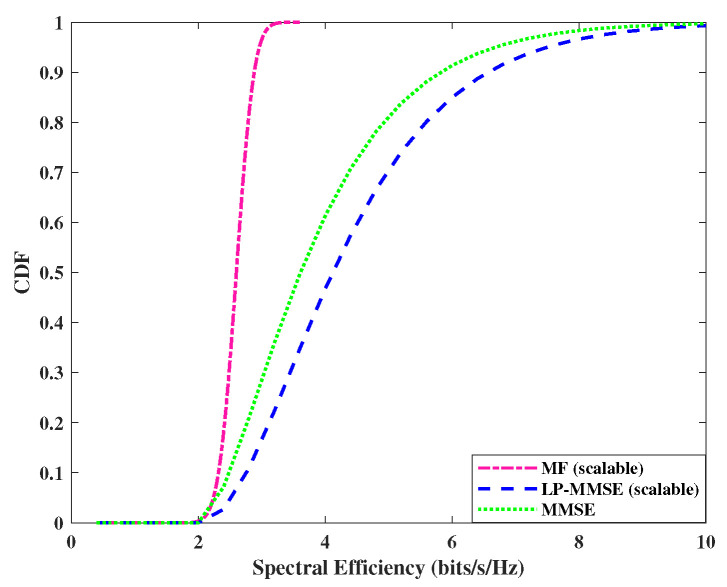
CDF of spectral efficiency for downlink, *N* = 200 APs and *M* = 4 antennas/AP.

The performance of downlink spectral efficiency is analyzed in Figure 7 and Figure 8, where we compare the performance of scalable, angle-based MF, MMSE, and scalable LP-MMSE at a fixed SNR level with DCC. The spectral efficiency of LP-MMSE is improved in comparison with the rest of the two schemes. The performances are compared in the downlink by utilizing max–min power control algorithm. As was the case with the uplink, for distributed scenario, N=200, M=4, the performance gap in spectral efficiency of MMSE Vs LP-MMSE and MF vs. LP-MMSE is decreased, i.e., LP-MMSE performance is slightly compromised against the MF and MMSE counterparts, whereas, the gap is increased in case of centralized scenario N=100, M=8. It is due to an increase in the number of antennas per AP, resulting in array gain. It is worth mentioning here that this array gain saturates at M≥32, as suggested in [5]. It is also evident from Figure 7 and Figure 8 that the performance gap between angle-based estimation schemes (LP-MMSE and MMSE) is not as high as in the uplink in both centralized (N=100, M=8) as well as distributed (N=200, M=4) scenarios. This is due to the fact that the higher power in the downlink signal gives leverage to MMSE, and its performance improves, however, spectral efficiency in MMSE is still lower than the LP-MMSE.

**Figure 9:**
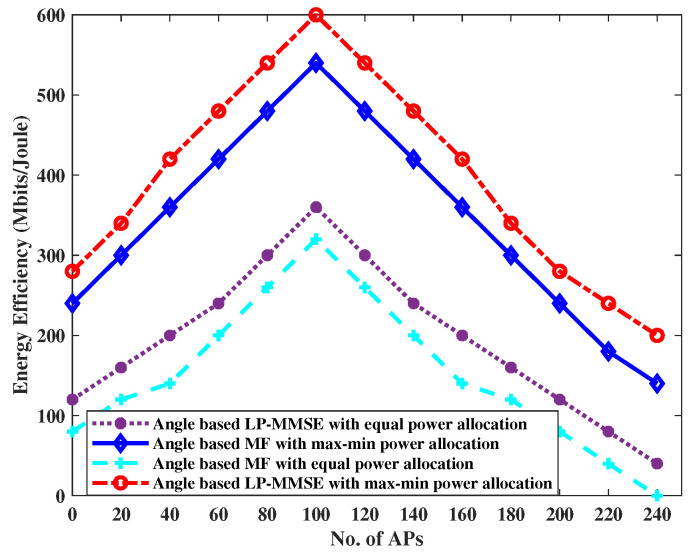
Comparison of energy efficiency for angle-based MF and angle-based LP-MMSE.

The energy efficiency versus the number of APs for an FDD-based cell-free network is compared and presented in Figure 9. We only considered the energy efficiency for scalable angle-based MF and LP-MMSE schemes with max–min power allocation versus equal power allocation algorithm. It is shown in Figure 9 that max–min power allocation algorithm gives a significant improvement in energy efficiency in both angle-based MF and angle-based LP-MMSE schemes. With the increase in the number of APs, there is a decrease in the number of antennas per AP, to keep MN constant over the coverage area. As a result, the energy efficiency starts to increase due to an increase in the number of APs per unit area. However, a further increase in the number of APs will result in a further reduction in the number of antennas per APs, causing spatial gain to decrease. Therefore, the energy efficiency degrades for the higher number of AP deployments in a cell-free framework due to an increase in backhaul power consumption, on the same lines as indicated by the results shown in [5]. It can be seen in the graph that for a given number of APs, e.g., N=100, max–min power control provides better energy efficiency, i.e., 520 Mbits/Joule for K=100 UEs, whereas, equal power algorithm gives 220 Mbits/Joule. The LP-MMSE with max–min power control shows the best performance, i.e., 580 Mbits/Joule vs. 280 Mbits/Joule with equal power allocation algorithm. It is worth mentioning here that an increase in the number of UEs such that $k\overset{}{\to }\infty$ will not effect the downlink energy efficiency as long as the combining/precoding vectors have finite complexity, which is true in our analysis of angle-based scalable MF and LP-MMSE schemes.

## 7. Conclusions

This work presents a scalable FDD-based cell-free massive MIMO system. Utilization of FDD in a cell-free setting can achieve reliable communication with low latency in applications with real-time communication. However, there are a few challenges associated with FDD utilization, such as higher computational complexity compared to its TDD counterpart. In addition, FDD-based systems do not include channel reciprocity; however, these systems encompass angular reciprocity, where AoA and AoD are the same in the uplink and downlink. We exploit this angular reciprocity to obtain the expressions for scalable angle-based beamforming and combining schemes in an FDD-based cell-free network. We utilized the concept of dynamic cooperation clustering for the computation of efficient and scalable beamforming and combining vector expressions. The DCC was applied to angle-based MF, angle-based LP-MMSE, and angle-based MMSE expressions. The performance of angle-based MF was compared with angle-based MMSE and angle-based LP-MMSE in terms of spectral and energy efficiency. The comparative analysis shows that LP-MMSE outperforms the MF and provides the best performance compared to the rest of the schemes. Future research directions may include the implementation of more efficient algorithms for joint initial access, AP clustering, and power control in an FDD cell-free framework. 

## Figures and Tables

**Table 1:** Comparison of proposed scalable angle-based FDD scheme with existing techniques.

Article	Scalable	Duplexing Mode	Precoding/ Combining	Power Allocation	Spectral Efficiency	Energy Efficiency
[4]	√	TDD	LP-MMSE, MMSE, MR	equal power	√	✗
[5]	✗	FDD	Angle-based MMSE, MF, and ZF	max–min, equal power	√	√
[27]	✗	TDD/FDD	L-MMSE	✗	√	✗
[28]	✗	NA	MR and MMSE	fractional, max–min, max-sum SE	√	✗
[29]	✗	TDD	fractional programming, convex–concave procedure	√	✗	
[30]	✗	TDD/FDD	TEAM-MMSE	✗	✗	✗
[31]	✗	FDD	angle-based ZF	non-convex QCQP	✗	✗
[34]	✗	TDD	hybrid precoding	✗	✗	✗
[35]	✗	TDD	joint MR and ZF	max–min	√	✗
[36]	✗	NA	MRC	✗	√	√
Proposed	√	FDD	Angle-based LP-MMSE, MMSE, and MF	max–min, equal power	√	√

**Table 2:** Comparison of computational complexity for estimation techniques.

Estimation Technique	Complexity of Estimation
Angle-based	Mlog(M)+VML
ESPRIT	$M^{3}+U_{i}N^{3}$
MUSIC	$\left(N^{3}M^{3}+K^{3}\right)$

**Table 3:** Comparison of computational complexity for estimation and combining schemes.

Technique	Complexity of Channel Estimation	Complexity of Precoding/Combining
Angle-based MMSE	($Mlog\left(M\right)+VML+M\tau_{p})K$ $|M_{k}|$	$\frac{{M|M|}_{k}log(M|M_{k}\left|)+M\right|M_{k}|}{2}K$ $+M|M_{k}\left|log(M\right|M_{k}\left|\right)$ $+\frac{\left(M\right|M_{k}{\left|\right)}^{3}-M\left|M_{k}\right|}{2}$
Angle-based LP-MMSE	$\left(Mlog\left(M\right)+VML+M\tau_{p}\right)$ $\sum_{n\in M_{k}}\left|A_{n}\right|$	$\frac{Mlog(M)+M}{2}\sum_{n\in M_{k}}|A_{n}|+\left(\frac{M^{3}-M}{3}+Mlog\left(M\right)\right)\left|M_{k}\right|$
Angle-based MF	$Mlog\left(M\right)+VML+M\tau_{p})$ $|M_{k}|$	-

**Table 4:** Simulation parameters for FDD-based cell-free framework.

System Parameters	Value
Coverage Area	2×2 km${}^{2}$
Bandwidth	100 MHz
Up/Downlink Frequency	49.8/50 GHZ
Transmit Power for Uplink Pilot	200 mW
Transmit Power of Payload in Uplink	200 mW
Transmit Power of Payload in Downlink	1 W
Angle Coherence Interval	200 Samples
Number of Monte Carlo Simulations	1000

## Data Availability

No new data were created or analyzed in this study. Data sharing is not applicable to this article.
